# Mild‐Temperature Catalyzed Hydrosilylation for Simplified Carbohydrate Functionalization of Porous Silicon Nanoparticles

**DOI:** 10.1002/chem.202402818

**Published:** 2024-12-16

**Authors:** Maria Grazia Nolli, Monica Terracciano, Ilaria Rea, Stefano D'Errico, Giuseppe Placido Mineo, Luca De Stefano, Gennaro Piccialli, Serena Riela, Giorgia Oliviero, Nicola Borbone

**Affiliations:** ^1^ Department of Pharmacy University of Naples Federico II via D. Montesano 49 80131 Naples Italy; ^2^ Naples Unit-National Research Council Institute of Applied Sciences and Intelligent Systems (ISASI) via P. Castellino 111 80131 Naples Italy; ^3^ Department of Chemical Sciences University of Catania Via A. Doria 6 95125 Catania Italy; ^4^ ISBE-IT University of Naples Federico II Corso Umberto I 40 80138 Naples Italy; ^5^ Department of Molecular Medicines and Medical Biotechnologies University of Naples Federico II via S. Pansini 5 80131 Naples Italy

**Keywords:** Porous silicon, Surface chemical modification, Nanoparticles, Hydrosilylation, Carbohydrates

## Abstract

Porous silicon is one of the most explored nanostructured materials in various biomedical applications owing to its remarkable properties. However, its inherent chemical instability mandates a robust surface modification procedure, and proper surface bioengineering is essential to ensure its effectiveness in the biomedical field. In this study, we introduce a one‐pot functionalization strategy that simultaneously stabilizes porous silicon nanoparticles and decorates their surface with carbohydrates through hydrosilylation chemistry, combining mild temperatures and a Lewis acid catalyst. This approach yielded a surface functionalization degree of 300 μmol g^−1^ in just 4 hours at 60 °C, significantly reducing both the prolonged reaction times and high temperatures typically associated with conventional hydrosilylation. Furthermore, this advancement opens the way for utilizing thermolabile molecules useful for surface bioengineering.

## Graphical abstract

A one‐pot hydrosilylation strategy is developed for the functionalization of porous silicon nanoparticles (PSiNPs) with allyl‐tetra‐O‐acetyl‐β‐D‐glucopyranoside (ATAG), using mild temperatures and ethylaluminium dichloride solution (EtAlCl_2_) as a catalyst. This efficient method reduces reaction times for passivating and bioengineering PSiNPs, enhancing their stability and potential for biomedical applications.

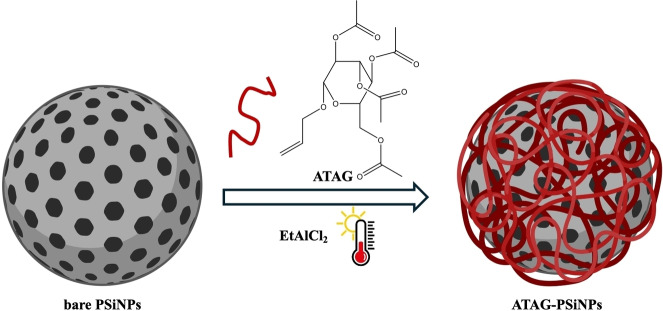

## 1 Introduction

Porous silicon (PSi) is a versatile material with intriguing properties that make it highly advantageous for various biomedical applications.^[1]^ A key feature is the ability to control the size, shape, and surface chemistry of its pores, both during and after fabrication.[^2^, ^3^] This precision in tailoring its properties distinguishes it from other commonly used mesoporous materials, enhancing its suitability for a range of biomedical uses.^[4]^ Notably, PSi is particularly effective for drug delivery (DD) due to its biocompatibility, high drug loading capacity, targeted delivery to specific cells, organs, or disease sites through proper surface bioengineering with targeting moieties such as peptides, sugars, proteins, and nucleic acids.[^1^, ^5^, ^6^, ^7^] Additionally, in vivo biodegradation of PSi results in the rapid clearance of non‐toxic products, primarily silicic acid (Si(OH)_4_).[^8^, ^9^, ^10^]

PSi is a top‐down material produced by the electrochemical etching of doped crystalline silicon wafers in anodization cells with hydrofluoric acid (HF).^[11]^ The freshly etched PSi consists of hydride‐terminated groups (Si−H, Si−H_2_, and Si−H_3_), making the PSi nanostructures highly reactive and unstable.^[12]^ Aging effects, resulting in the uncontrolled growth of native oxide and degradation of the PSi matrix in alkaline or aqueous environments, significantly impact the material's physicochemical properties, making its practical use difficult.^[13]^ Moreover, the capability to precisely bioengineer the PSi surface is crucial for enhancing its performance and functionality in the biomedical field.[^14^, ^15^, ^16^] For example, for DD applications, proper chemical surface modifications of PSi nanoparticles (PSiNPs) can enhance biocompatibility, enable targeted delivery to specific cells or tissues, improve stability and dispersibility, facilitate controlled drug release, evade immune recognition, and introduce multifunctionalities such as imaging and diagnostics.[^17^, ^18^, ^19^, ^20^, ^21^]

Numerous surface chemical passivation/functionalization strategies for PSi structures have been developed, typically involving silicon‐oxygen (Si−O) or silicon‐carbon (Si−C) bond formation and the introduction of functional coupling linkers (−NH_2_, −COOH, −SH, etc.) essential for surface decoration with bioprobes. However, developing efficient bioengineered PSi structures for biomedical applications often requires time‐consuming, multi‐step chemical procedures. Some of these protocols are not suitable for all molecules, particularly thermolabile ones, complicating the functionalization process.[^1^, ^5^, ^17^]

In this study, we present an advancement in the functionalization of PSiNPs by combining the traditionally separate processes of passivation and functionalization into a single one‐pot reaction. This approach utilizes mild temperature conditions and an appropriate Lewis acid catalyst, which not only reduces reaction times but also expands the method's applicability to thermolabile molecules.^[22]^

As a model for this strategy, we selected allyl‐tetra‐*O*‐acetyl‐β‐D‐glucopyranoside (ATAG), chosen for its promising potential in biomedical applications.^[6]^

Surface decoration with carbohydrates offers several advantages for DD purposes. Sugar molecules improve the stability and dispersibility of drug‐loaded nanoparticles (NPs) and enhance biocompatibility and biodegradability, minimizing potential toxicity and side effects.^[23]^ Moreover, carbohydrates can act as ligands for specific receptors (e. g., GLUT 1 and GLUT 3) expressed on the surface of tumor cells and metabolically active tissues, making glucose‐functionalized NPs ideal candidates for targeted DD.[^24^, ^25^, ^26^]

We investigated a range of reaction conditions to identify the optimal approach, thus establishing a foundation for future applications, particularly in DD. The most effective strategy was thoroughly evaluated using a combination of qualitative and quantitative techniques, including solution‐phase proton nuclear magnetic resonance (^1^H NMR), micro Fourier transform infrared spectroscopy (microFT‐IR), dynamic light scattering (DLS), transmission electron microscopy (TEM), and thermogravimetry (TGA).

## Materials and Methods

### Chemicals

Hydrofluoric acid (HF), water, ethanol (EtOH), isopropanol (IPA), methanol (MeOH), chloroform (CHCl_3_), d‐chloroform (CDCl_3_), anhydrous toluene (Tol‐dry), ethylaluminium dichloride solution (EtAlCl_2_ 1.0 M in hexane), cerium (IV) sulfate, and Phosphate‐Buffered Saline 1x (PBS) were purchased from Sigma Aldrich (Merck KGaA, Darmstadt, Germany). Sulphuric acid 96 % was purchased from Carlo Erba Reagents S.r.l. (Cornaredo, Italy). Allyl‐tetra‐*O*‐acetyl‐β‐D‐glucopyranoside (ATAG) was purchased from Biosynth S.r.o. (Nobelova, Bratislava, Slovakia).

### Bare PSiNPs Fabrication

The PSi structure was fabricated by electrochemical etching of n^+^ type crystalline silicon (0.01–0.02 Ω cm resistivity, <100> oriented and 600–550 μm in thick) doped with antimony. Before etching, the silicon substrate was immersed for 5 min in 25 mL HF, 70 mL water, and 130 mL ethanol 96° (HF solution) at room temperature to remove the native oxide layer (refreshing procedure).

After the refresh, the crystalline silicon was washed with IPA and air‐dried. Then, it was placed in the electrolytic cell where it initially underwent the electropolishing regime in which a current density of 220 mA was applied for 200 ms, with a pause of 5 s. This current density was applied for 20 cycles. A current density of 20 mA was then applied for 135 cycles of 200 ms pulses (5 s pause between cycles) to get a single layer of mesoporous silicon with 61 % porosity, thickness (L) of 6 μm, and pore dimension between 2 and 50 nm. Finally, detaching was carried out with the same conditions as electropolishing, and the resulting chip (6 μm thickness) was removed from the cell, placed in 5 mL of IPA, and sonicated at RT for 5 min in an DU‐32 digital bath ultrasonicator (Argo Lab, Carpi, Italy). At the end, the membrane was sonicated for 12 h at the maximum power of 40 kHz at the controlled temperature of 15 °C, achieving bare PSiNPs which were quantified by weighing on an ABJ 220–4NM analytical balance (Kern & Sohn GmbH, Balingen, Germany). HF is a highly hazardous and corrosive chemical that requires strict adherence to safety protocols during use. In this study, all procedures involving HF were conducted in a certified fume hood to prevent inhalation of fumes. Trained personnel wore appropriate personal protective equipment (PPE), including acid‐resistant gloves, safety goggles, and laboratory coats. Emergency safety measures, including access to calcium gluconate gel for immediate treatment in case of accidental exposure, were in place throughout the experiments.

### PSiNPs Characterization

#### Micro Fourier Transform Infrared Spectroscopy (Micro‐FTIR)

Micro‐FTIR spectra of PSiNPS were recorded on a Nicolet iN10 infrared spectrophotometer (Thermo Fisher Scientific, Waltham, MA, USA) in transmittance mode using 64 scans per acquisition. The spectra were collected in the range of 4000–500 cm^−1^ with a resolution of 4 cm^−1^. All samples (about 800 μg suspended in 10 μL of IPA) were deposited on a gold surface and air‐dried.

#### Dynamic Light Scattering (DLS)

50 μL samples of either 2.5 mg bare PSiNPs or ATAG‐modified PSiNPs (ATAG‐PSiNPs) in 5 mL of IPA were suspended in 950 μL of water and subjected to size distribution and ζ‐potential measurements at 25 °C on a Zetasizer Nano ZS instrument (Malvern Panalytical, Malvern, UK) equipped with a HeNe laser (633 nm, 173° fixed scattering angle).

#### ^1^H NMR

^1^H NMR data were collected at 25 °C on a Bruker Avance Neo 600 spectrometer (Bruker BioSpin, Billerica, MA, USA), operating at 600 MHz and equipped with a triple resonance cryoprobe. The NMR samples were prepared as following: ATAG (2 mg) in 600 μL CDCl_3_; bare PSiNPs (1 mg) in 250 μL CDCl_3_; ATAG‐PSiNPs (2.5 mg) in 600 μL CDCl_3_. The spectra were processed using the Mnova 14.3.0 software suite (Mestrelab Research, Santiago de Compostela, Spain) and referenced to the residual solvent signal (7.26 ppm CHCl_3_).

#### Transmission Electron Microscopy (TEM)

The morphology of dried bare PSiNPs and ATAG‐functionalized PSiNPs (ATAG‐PSiNPs) (50 μg mL^−1^) dropped on a carbon‐coated copper grid was assessed by transmission electron microscopy using a Jeol JEM‐1400 instrument by Jeol Ltd (Tokyo, Japan).

### Hydrosilylation of Bare PSiNPs with ATAG

#### Stability Test of ATAG and PSiNPs

The stability of ATAG at 40 °C and 60 °C was assessed by cerium solfate TLC monitoring. Briefly, 6 mg of sugar was solubilized under dry condition in 1200 μL of tol‐dry, mimicking the hydroxylation reaction without using PSiNPs. This approach allowed us to evaluate the reaction conditions and confirm the stability of the sugar derivative prior to its conjugation with the NPs. For the stability test at 40 °C, the sample was taken at times 0 and 5 h; for the test at 60 °C, the sample was collected at times 0, 3, and 10 h. Collected samples were loaded on aluminium oxide TLC plates which were eluted using an 8 : 2 (10 mL) petroleum ether/ethyl acetate mixture. After the migration, the plates were air‐dried and immersed in a solution of cerium sulfate (6.5 g cerium ammonium sulfate, 3 mL H_2_SO_4_ 98 %, and 50 mL water). Finally, the plates were visualized by heating at 200 °C on a hot plate. NMR and Electrospray Mass Spectrometry (ESI‐MS) analysis, using a 4000 QTRAP mass spectrometer (ThermoFisher Scientific, Waltham, MA, USA), were also carried out to confirm the stability of ATAG at 40 and 60 °C.

The stability of both bare and modified NPs was evaluated by incubating them in PBS at pH 7.2 at 37 °C under constant agitation. The stability was assessed by monitoring changes in the hydrodynamic diameter of the NPs over time, up to 24 hours.

#### Hydrosilylation Reaction

6 mg of ATAG were dissolved in 600 μL of tol‐dry (solubility 10 mg mL^−1^) and added to 2.5 mg of bare PSiNPs, dispersed in 600 μL of tol‐dry, in the presence of EtAlCl_2_ 1 M in hexane (14 μL) as catalyst.[^27^, ^28^, ^29^] The first reaction was carried out for 7 hours at 60 °C, followed by overnight incubation at room temperature (RT), while the second reaction was conducted for 4 hours at 60 °C. Both reactions were performed under dry conditions and an argon atmosphere. After the hydrosilylation reaction, the mixture was centrifuged three times at 15,000 rpm for 30 min using a SCI24R refrigerated micro‐centrifuge (Scilogex, Rocky Hill, CT, USA), and the supernatant was removed and replaced once with tol‐dry, once with MeOH, and once with CHCl_3_ to remove the sugar in excess.

### Thermogravimetric Analyses (TGA)

Thermogravimetric analyses on bare and ATAG‐PSiNPs were performed under air atmosphere (60 mL min^−1^) in the temperature range 50–800 °C (thermal ramp of 10 °C min^−1^) using a TGA 7 thermogravimetric analyzer equipped with a TAC7/DX thermal analysis controller (Perkin Elmer, Waltham, MA, USA).

## 2 Results and Discussion

### 2.1 Optimization Studies of Bare PSiNPs Fabrication

Producing PSi nanostructures with uniform sizes presents several challenges due to variability in the synthesis process, potentially resulting in NPs of different sizes even under identical conditions. Therefore, precise optimization of synthesis parameters is crucial to overcoming these challenges.^[29]^

In this study, we developed a standardized protocol for obtaining NPs of specific sizes, achieving samples with acceptable homogeneity.

We initially calculated the theoretical yield of PSiNPs obtainable from a 6 μm‐thick free‐standing PSi membrane produced by a single electrochemical etching of crystalline silicon. This theoretical value was confirmed analytically by weighing the PSi membrane obtained from the etching process (see Equations (1), (2), and (3) in the Supporting Information). The experimental measurements and theoretical calculations closely matched, confirming that approximately 0.5 mg of NPs can be obtained from a 6 μm‐thick free‐standing PSi membrane.

To obtain reproducible NPs samples with suitable hydrodynamic diameters, we tested various sonication protocols to determine the optimal sonication condition.

**Figure 1:**
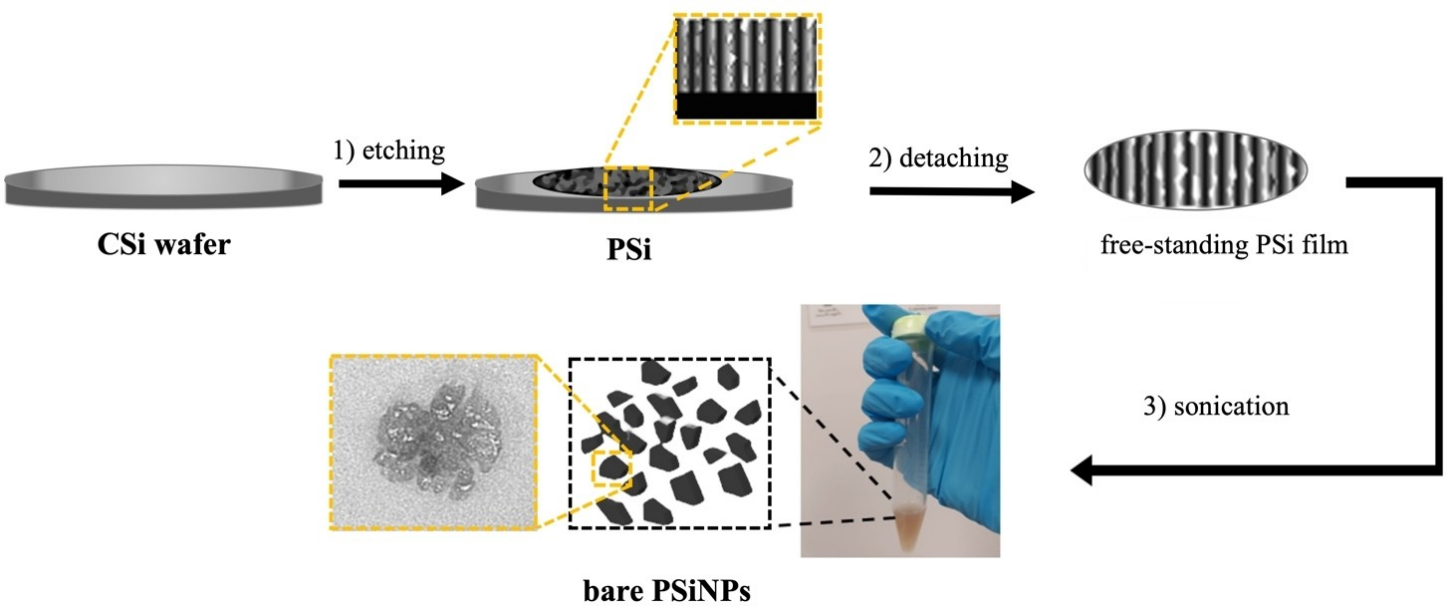
Representation of bare PSiNPs fabrication: A 2 cm^2^ area of a crystalline silicon wafer underwent electrochemical etching to produce a PSi layer. Detaching the layer released a 6 μm‐thick film, which was then sonicated to produce bare PSiNPs.

We demonstrated that dispersing the obtained free‐standing PSi in dry IPA solvent and treating it to a 12 hour bath sonication at a maximum power of 40 kHz at a controlled temperature of 15 °C allowed us to produce bare PSiNPs sizing 480±70 nm (see Figure 1 and Figure S1).

### 2.2 Hydrosilylation of Bare PSiNPs with ATAG

Hydrosilylation is a widely used technique for the passivation and functionalization of PSi structures based on the formation of silicon‐carbon (Si−C) bonds.[^30^, ^31^] With this chemical strategy, it is possible to introduce functional groups useful for binding biomolecules, polymers, metals, or other substances onto the PSi surface, thus introducing new properties or functionalities.^[32]^

Despite its extensive use, hydrosilylation of PSi may present some drawbacks, such as long reaction times at high temperatures. To overcome these challenges, we developed a one‐pot chemical protocol using allyl‐modified sugar (ATAG) as the hydrosilylation agent, enabling the efficient functionalization of bare PSiNPs within shorter reaction times and under mild temperature conditions. ATAG was chosen as a model molecule for functionalizing NP surfaces due to its significant biomedical potential. As a primary metabolite, glucose functionalization could enhance NPs biocompatibility, minimizes toxicity, and facilitates targeted delivery to tumor cells and metabolically active tissues via GLUT‐1 receptors. Additionally, it could provide a protective barrier for encapsulated drugs, improve solubility in aqueous environments, and offers functional groups for further chemical modifications, significantly expanding its applicability in targeted DD.[^24^, ^25^, ^26^]

**Figure 2:**
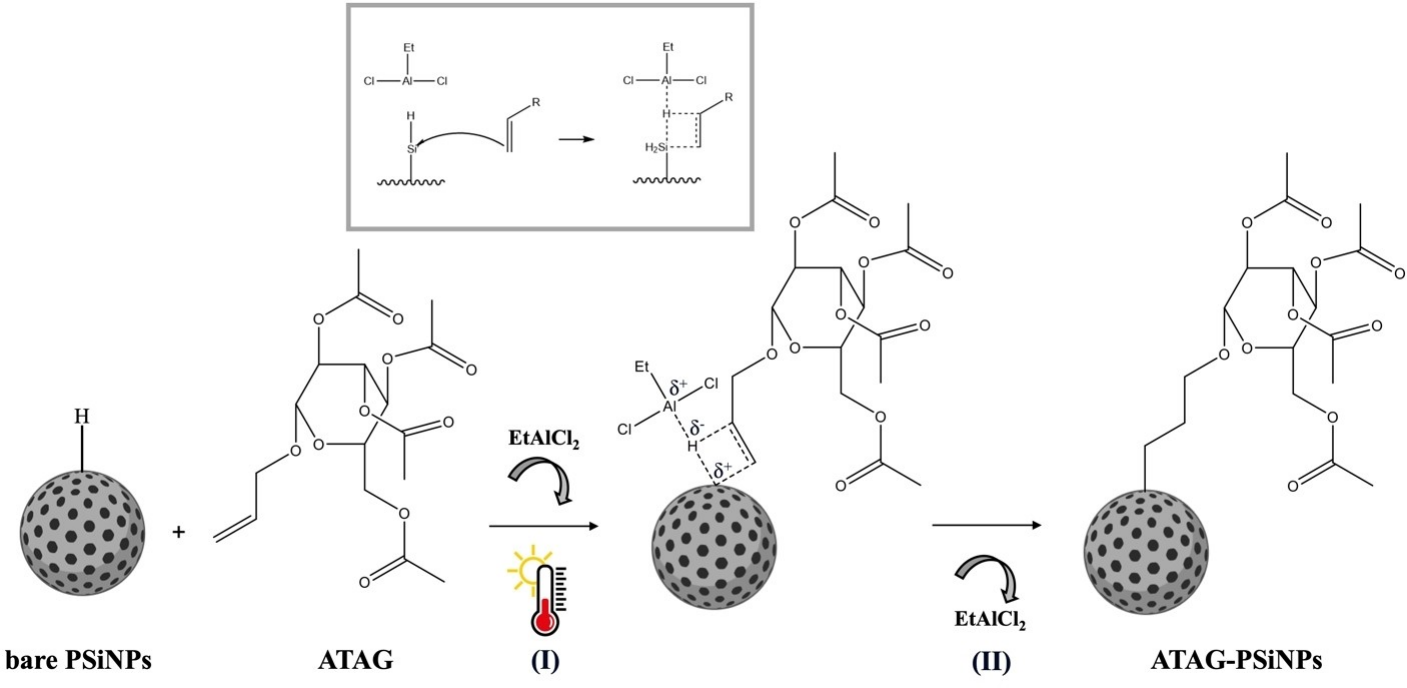
Schematic representation of the hydrosilylation of bare PSiNPs. The reaction is carried out with EtAlCl_2_ as the catalyst and under mild temperature (I, 40 or 60 °C), thus achieving a reaction intermediate (II), and then the ATAG‐PSiNPs.

Herein, we exploited the synergistic effect achieved by incorporating EtAlCl_2_, a commonly used Lewis's acid catalyst in hydrosilylation, while employing mild temperatures (40 or 60 °C) to accelerate the reaction kinetics (Figure 2). We chose EtAlCl_2_ as the catalyst because it was able to strip a proton from the Si−H bond, polarising it and giving the silicon atom a partial positive charge. This step facilitates the reactivity of the alkene, allowing the attack of the allyl group on the silicon atom to generate the Si−C bond through the formation of a four‐centred intermediate (see the insert in Figure 2).^[33]^

To assess the feasibility of the proposed reaction, we performed stability tests on ATAG under conditions simulating the intended reaction temperatures of 40 and 60 °C, both of which are below the sugar's melting point of 89 °C. ATAG samples were collected at three time points: 0 hours (T0, as control), 3 hours (T_3h_), and 10 hours (T_10h_) and then analysed with TLC and a cerium (IV) sulfate assay.

The analysis revealed that all samples migrated with the same Retention Factor (Rf), indicating that the sugar did not undergo degradation at these temperatures. We employed the cerium (IV) sulfate assay to visualize the position of sugar on TLC plate. In this assay upon exposure of the TLC plate to a temperature of 200 °C, a carbonization reaction occurred, further influencing the observed colour change (Figure S2). However, to validate this data, we also conducted both mass spectrometry and NMR characterizations (Figure S3 and Figure S4).

In Figure S3, the mass spectrum of ATAG at T_0_, used as a control, showed a peak at 411 Da, due to the formation of a sugar adduct with sodium. The same peak at 411 Da was also found in the ATAG spectrum both at 40 °C and 60 °C after 10 hours, confirming the stability of ATAG at these temperatures. Moreover, the spectrum at 60 °C showed a second peak at an m/z value of 798.5 Da, characteristic of the formation of the ATAG dimer. The NMR spectra validated the stability of ATAG at both 40 °C and 60 °C, as evidenced by the ^1^H proton signals of treated sugars remaining consistent with those of the untreated sample (Figure S4).

We opted for the higher temperature tested (60 °C) to stay closer to the conventional hydrosilylation conditions, which traditionally start at temperatures around 110 °C to kick off the reaction.^[6]^ However, such high temperatures were no longer necessary for the reaction acceleration due to the catalyst.

The as‐etched bare PSiNPs were dissolved in ATAG tol‐dry solution in the presence of EtAlCl_2_ at 60 °C, under stirring. The reaction proceeded for seven hours at 60 °C and overnight at RT in argon atmosphere. Periodic sampling of NPs was performed at different times (4 h, 7 h, and 17) to monitor the progression of the reaction kinetics. Before NPs characterization, each aliquot underwent meticulous washing with different solvents (tol‐dry, MeOH, IPA) to remove excess of adsorbed sugar and finally re‐suspended in IPA. The washing supernatants were analysed by cerium (IV) sulfate assay to detect any remaining residues of ATAG. During the initial wash, an excess of sugar triggered a carbonization reaction when the TLC plate was exposed to a temperature of 200 °C. Conversely, in the final wash no traces of ATAG remained, as typical charring stain of carbonization process did not appear (Figure S5).

**Figure 3:**
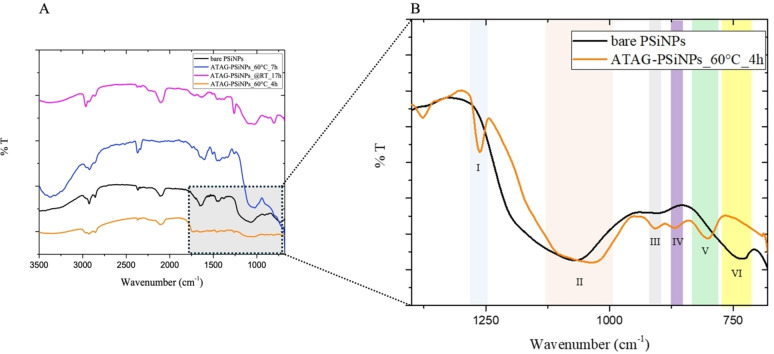
**A**) Superimposition of microFT‐IR spectra of bare PSiNPs (in black), ATAG‐PSiNPs at 60 °C for 4 hours (in orange) and 7 hours (in sky blue), and at @RT for 17 hours (in magenta); B) Superimposition of microFT‐IR spectra of bare PSiNPs (in black) and ATAG‐PSiNPs (in orange): (I) 1260 cm^−1^, CH_x_ symmetric stretching; (II) 1180–1050 cm^−1^, Si−O−Si bonds; (III) 910 cm^−1^, SH_2_ scissor bending; (IV) 867 cm^−1^, Si−CH_x_ stretching; (V) 802 cm^−1^, Si−C; (VI) 733 cm^−1^, Si−H wag.

To assess the efficacy of surface modification on bare PSiNPs, we conducted microFT‐IR analyses (Figure 3).

Comparison of the NPs spectra before and after chemical treatments confirmed almost complete passivation/modification of bare PSiNPs, evidenced by the presence of characteristic peaks of Si−C covalent bonds and sugar: C−Hx symmetric stretching signals at 1260 cm^−1^, 802 cm^−1^, and 867 cm^−1^, and the subsequent disappearance of the Si−H signal at 733 cm^−1^. However, all FTIR spectra exhibited a weak characteristic peak of Si−O−Si stretching mode at 1090–1100 cm^−1^, likely attributed to spontaneous aging of PSi powders during handling.[^34^, ^35^, ^36^] Additionally, peaks related to the stretching of the −OH group (3300–3200 cm^−1^) and C−H (2920–2850 cm^−1^) of residual IPA, used as the solvent phase, were observed both in bare PSiNPs and modified NPs due to an incomplete solvent evaporation. Interestingly, all modified NPs showed nearly identical spectra, confirming the success of the modification and the remarkable effectiveness achieved within just 4 h of reaction. This condition was selected as the optimal, offering a significant advantage over conventional longer reaction times.

**Figure 4:**
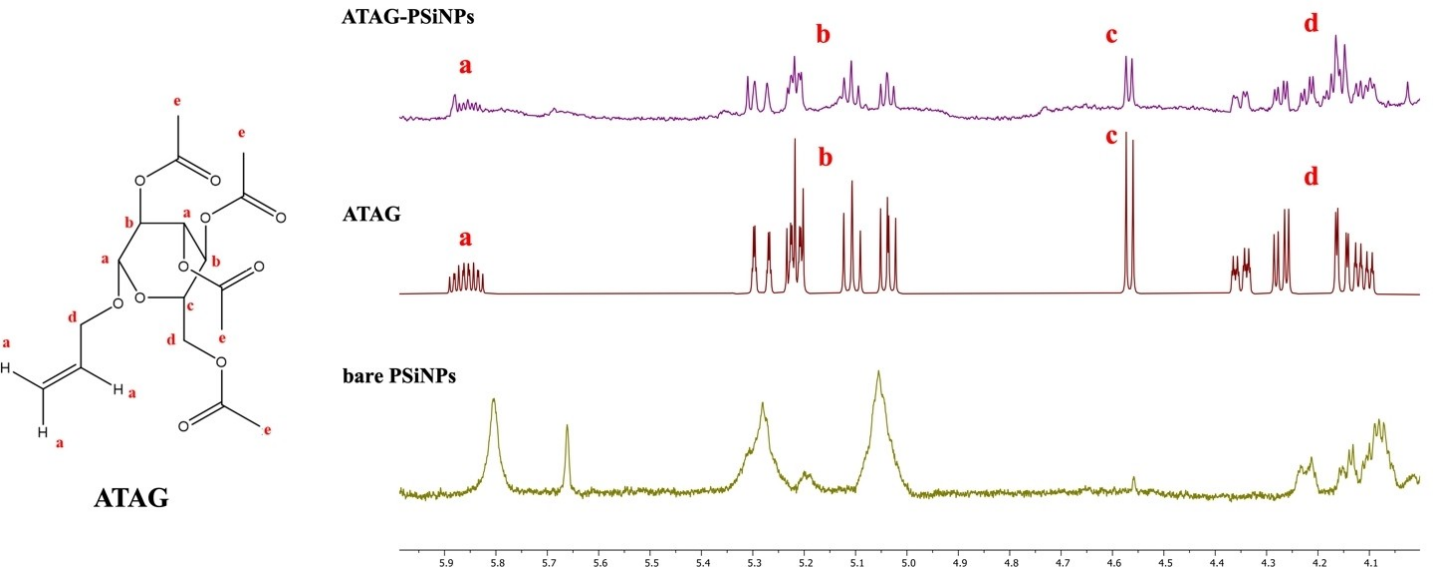
Superimposition of spectra of bare PSiNPs (in green), ATAG (in magenta), and ATAG‐PSiNPs (in violet). The characteristic peaks of the ATAG moiety protons are shown as Ha 5.31–6.07 ppm, Hb 5.26–5.56 ppm, Hc 4.55 ppm, Hd 4.0–4.38 ppm.

NMR spectroscopy was used to confirm the conjugation of the sugar derivative to the PSiNPs surface. In Figure 4, the ^1^H NMR spectrum of ATAG‐PSiNPs dispersion shows the characteristic peaks (a–d) of the ATAG moiety protons (see Figure S6 for peaks a–e). In particular, new peaks appeared in the NMR spectra after functionalization, corresponding to the protons of the grafted glucose moiety (Ha 5.31–6.07 ppm, Hb 5.26–5.56 ppm, Hc 4.55 ppm, and Hd 4.0–4.38 ppm), which were absent in the spectra of bare NPs. These spectral changes, along with the comparison of pre‐ and post‐functionalization spectra, confirm the successful conjugation of glucose to the NPs. The ^1^H NMR spectra and chemical shifts of the free ATAG and bare PSiNPs are also shown for reference in Figure 4 and Table 1, respectively.

**Table 1:** Proton chemical shifts of ATAG sugar molecules.

Atom	Δδ (ppm)
Ha	5.31–6–07
Hb	5.26–5.56
Hc	4.55
Hd	4.0–4.38
He	2.02–2.04

**Figure 5:**
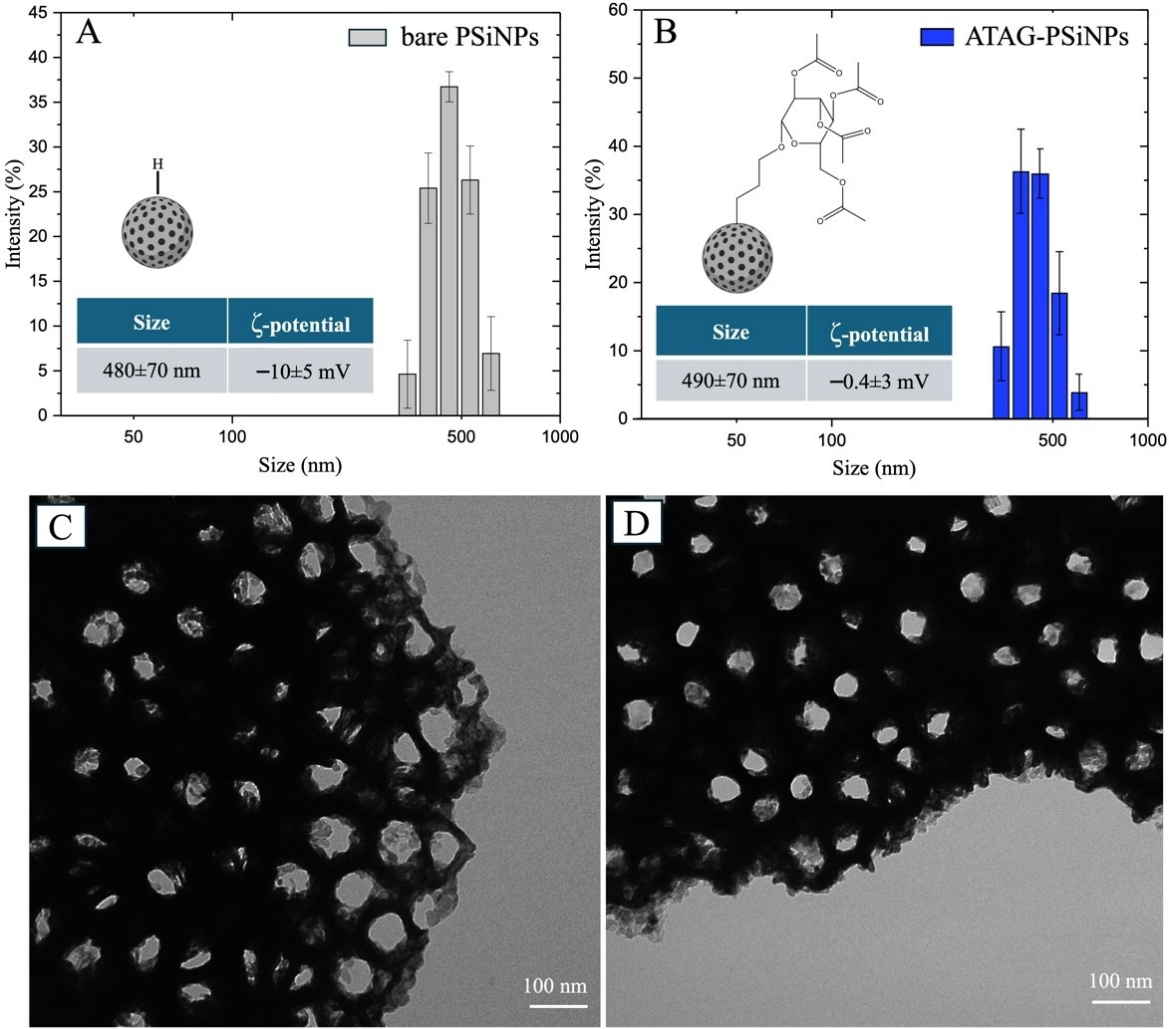
Schematic representation of the hydrodynamic radius and zeta potential of A) bare PSiNPs and B) ATAG‐PSiNPs. The error bars represent the standard deviation (SD) of three independent measurements (n=3). Representative image of TEM characterization of micrometric bare (C) and ATAG‐PSi particles (D).

Then, we evaluated the hydrodynamic diameter and the zeta potential of the PSiNPs before and after their chemical modification by DLS. We did not observe increase in the NPs’ size (both were similar within the error, being from 480±70 nm to 490±70 nm). We found that due to the reduction in surface repulsion forces, the zeta potential increased from −10±5 mV to −0.4±3 mV, thus validating the hydrosilylation process (Figure 5A and B). The ATAG resulted in a neutral charge due to the acetyl groups (−COCH3) used to cap the hydroxyl groups (−OH) of the sugar to prevent undesired reactions. We performed a preliminary stability test on the modified PSiNPs in PBS medium (pH 7.2) at 37 °C over a 24 hour period. The results revealed that the modified NPs maintained stability throughout the entire duration, while bare NPs exhibited significant instability, characterized by size reduction (Figure S7). Remarkably, the modified NPs demonstrated superior stability compared to those functionalized via the traditional hydrosilylation method with undecylenic acid (18 h at 110 °C), which retained stability for only 6 hours.^[6]^ This enhanced performance is attributed to the ability of the developed method to achieve faster and efficient Si−H to Si−C bond conversion, leading to improved stability.

Finally, we examined micrometric PSi structures by TEM imaging to better evaluate the morphological alterations resulting from the one‐pot modification (Figure 5C and D).

The analysis revealed the persistent irregular shape and porous morphology of the PSi microstructures, with pore diameters ranging from 10 nm to 50 nm. This underscores that the employed strategy did not adversely impact the particles’ morphology.

### 2.3 Quantification of the Reaction Yield by TGA

Thermogravimetric analysis (TGA) was performed to gain information regarding the thermal stability of PSiNPs before and after ATAG surface modification and the conjugation of organic species on the NPs’ surface. Under air atmosphere, both samples exhibited gradual degradation starting from approximately 400 °C, with a progressive weight loss observed between around 100–400 °C, stabilizing at a final mass value of 88.78 %.

**Figure 6:**
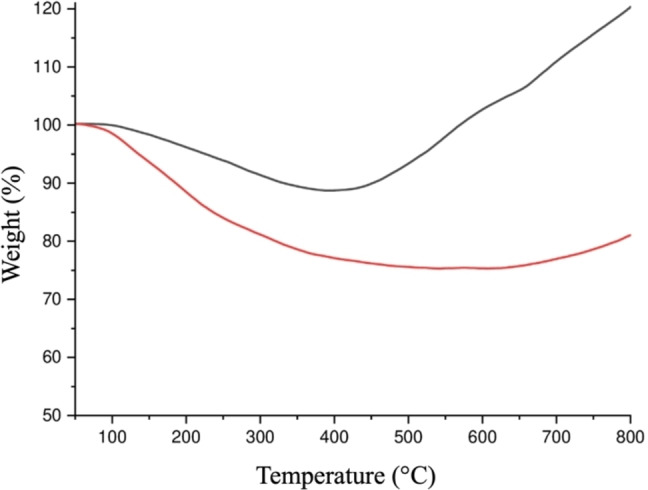
Thermoanalytical curves of bare PSiNPs (black line) and ATAG‐PSiNPs (red line) samples in the range of 50–800 °C.

Upon heating up to 800 °C in an air atmosphere, bare PSiNPs showed a weight increase of 31.72 % from 400 to 800 °C due to the partial oxidation of PSiNPS (Figure 6, black line). In contrast, ATAG‐PSiNPs exhibited a different trend in the TG curve compared to bare PSiNPs. The weight percentage decreased to a minimum value of 77.23 wt % at approximately 400 °C, due to the complete burn‐off of the organic moieties on the NPs’ surface, followed by a slight increase of only 4 % in the 400–800 °C range. Notably, after functionalization, the ATAG‐PSiNPs sample demonstrated enhanced stability toward oxidation compared to the bare PSiNPs (Figure 6, red line). Based on TGA analysis, 400 °C is assumed to be the critical temperature for determining the ATAG/ATAG‐PSiNPs ratio.

The weight loss observed in the TGA analysis, attributed to the degradation of the sugar derivative, was used to determine the weight percent (11.5 w‐%) of the grafted sugar on the NPs by using the Equation (4) (see Supporting Information) corresponding to a functionalization degree of 0.30 mmol g^−1^.

## 3 Conclusions

Porous silicon (PSi) stands out as a highly versatile and advantageous material for biomedical applications, particularly in drug delivery, due to its tunable porosity, biocompatibility, and biodegradability. The ability to precisely control the size, shape, and surface chemistry of PSi enhances its performance over other mesoporous materials, enabling high drug loading capacity and targeted delivery through surface bioengineering. However, the inherent instability and reactivity of freshly etched PSi, along with aging effects, pose significant challenges to its practical application.

To address these challenges, we developed a one‐pot functionalization strategy that stabilizes PSi nanoparticles (PSiNPs) using hydrosilylation chemistry while simultaneously decorating their surface with carbohydrates such as allyl‐tetra‐O‐acetyl‐β‐D‐glucopyranoside. This approach utilizes mild temperatures and a Lewis acid catalyst, significantly reducing reaction time and avoiding the high temperatures required in traditional methods. To optimize the chemical strategy, we employed various conditions and validated the best one using ^1^H NMR, microFT‐IR, DLS, TEM, and TGA.

The proposed method simplifies the passivation and bioengineering of PSiNPs, enhancing efficiency and versatility, particularly for thermolabile molecules.

This chemistry‐driven study provides a solid foundation for advancing glucose‐functionalized PSiNPs as promising candidates for drug delivery (DD). Future work will focus on refining the functionalization strategy, evaluating stability under various conditions, and assessing NPs performance in diverse biological environments, ultimately enabling the development of innovative, targeted DD systems tailored to specific therapeutic needs.

## Consent for Publication

All authors have approved the manuscript and agree for the submission.

## Author Contributions

M.G. Nolli: formal analysis, investigation, data curation, methodology, writing original draft. M. Terracciano: conceptualization, formal analysis, supervision, writing — original draft, writing — review and editing. I. Rea: conceptualization, formal analysis, supervision, writing — original draft, writing — review and editing. S. D'Errico: data curation, methodology, resources. G. Placido Mineo: data curation, methodology, resources. L. De Stefano: conceptualization, formal analysis, supervision, writing — original draft, writing — review and editing. G. Piccialli: data curation, methodology, resources. S. Riela: conceptualization, formal analysis, supervision, writing — original draft, writing — review and editing. G. Oliviero: conceptualization, formal analysis, supervision, writing — original draft, writing — review and editing. N. Borbone: conceptualization, formal analysis, supervision, writing — original draft, writing — review and editing.

## Conflict of Interests

There are no conflicts to declare.

4

## Supporting information

As a service to our authors and readers, this journal provides supporting information supplied by the authors. Such materials are peer reviewed and may be re‐organized for online delivery, but are not copy‐edited or typeset. Technical support issues arising from supporting information (other than missing files) should be addressed to the authors.

Supporting Information

## Data Availability

The data that support the findings of this study are available from the corresponding authors upon reasonable request.
