# Supplementary material for: Mild‐Temperature Catalyzed Hydrosilylation for Simplified Carbohydrate Functionalization of Porous Silicon Nanoparticles
Source: Chemistry. 2024 Dec 16;31(2):e202402818. doi: 10.1002/chem.202402818 (PMC11724234; doi:10.1002/chem.202402818)
Supplement: Supplementary file 1 — Supporting Information [file CHEM-31-e202402818-s001.pdf]

Chemistry–A European Journal

Supporting Information

Mild-Temperature Catalyzed Hydrosilylation for Simplified Carbohydrate Functionalization of Porous Silicon Nanoparticles

Maria Grazia Nolli, Monica Terracciano,* Ilaria Rea, Stefano D'Errico, Giuseppe Placido Mineo, Luca De Stefano, Gennaro Piccialli, Serena Riela, Giorgia Oliviero,* and Nicola Borbone

Supporting Information

MILD-TEMPERATURE CATALYZED HYDROSILYLATION FOR SIMPLIFIED CARBOHYDRATE FUNCTIONALIZATION OF POROUS SILICON NANOPARTICLES

Maria Grazia Nolli[1], Monica Terracciano[1, *], Ilaria Rea[2], Stefano D'Errico[1], Giuseppe Placido Mineo[3], Luca De Stefano[2], Gennaro Piccialli[1,4], Serena Riela[3], Giorgia Oliviero[4,5, *], and Nicola Borbone[1,4]

[1]Department of Pharmacy, University of Naples Federico II, via D. Montesano 49 - 80131 Naples, Italy

[2]Institute of Applied Sciences and Intelligent Systems (ISASI), Naples Unit-National Research Council, via P. Castellino 111 - 80131 Naples, Italy

[3]Department of Chemical Sciences, University of Catania, Via A. Doria 6 - 95125 Catania, Italy

[4]ISBE-IT, University of Naples Federico II, Corso Umberto I 40 - 80138 Naples, Italy

[5]Department of Molecular Medicines and Medical Biotechnologies, University of Naples Federico II, via S. Pansini 5 - 80131 Naples, Italy

*Corresponding authors: monica.terracciano@unina.it; golivier@unina.it

Equation 1: $Vm = \pi r^2 h$

To theoretically calculate the volume of the membrane for each chip, we used Equation 1.

Given that an O-ring with a radius (r) of 0.55 cm and a membrane height (h) of 6 µm was used in the fabrication of PSi, we obtained a volume of $5.7.10^{-4}$ cm^3.

Next, we calculated the volume of the PSi considering the material's porosity (61%) and the occupied volume fraction (V_o 39%) as shown in Equation 2.

Equation 2: $VSi = Vm\ x\ Vo$ = $5.7.10^{-4}$ cm^3 x 0.39 = $2.2.10^{-4}$ cm^3

Finally, we calculated the mass (M) of bare PSiNPs using Equation 3, considering the density (ρ) of crystalline silicon.

Equation 3: $M = \rho\ x\ VSi$ = 2.33 g/cm^3 x $2.2.10^{-4}$ cm^3 = $5.2.10^{-4}$ g

Equation 4:

$$w\% = \frac{\text{(Weight loss attributed to grafted sugar)}}{\text{(Initial weight of functionalized nanoparticles)}} x\ 100$$

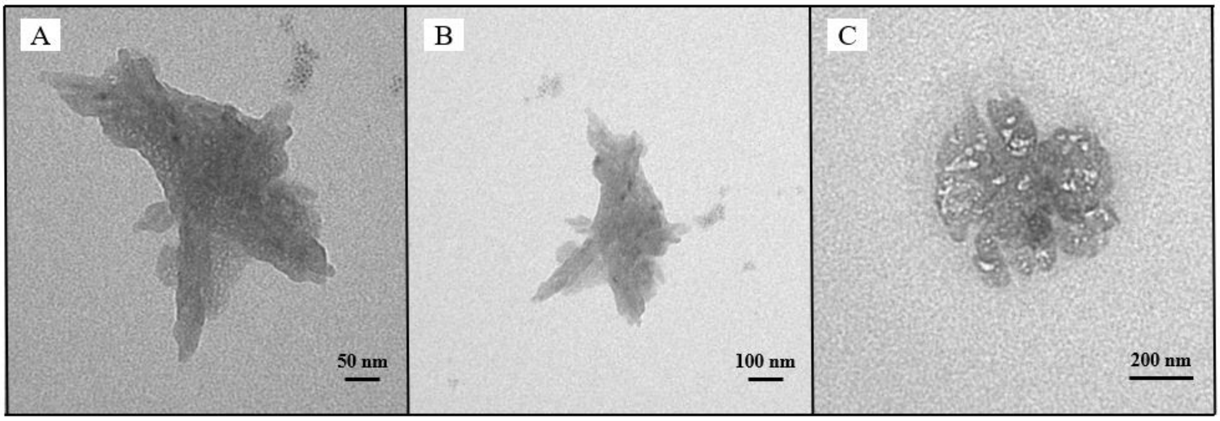

Fig. S1 TEM characterization of bare PSiNPs; A) Resolution of 50 nm; B) Resolution of 100 nm; C) Resolution of 200 nm.

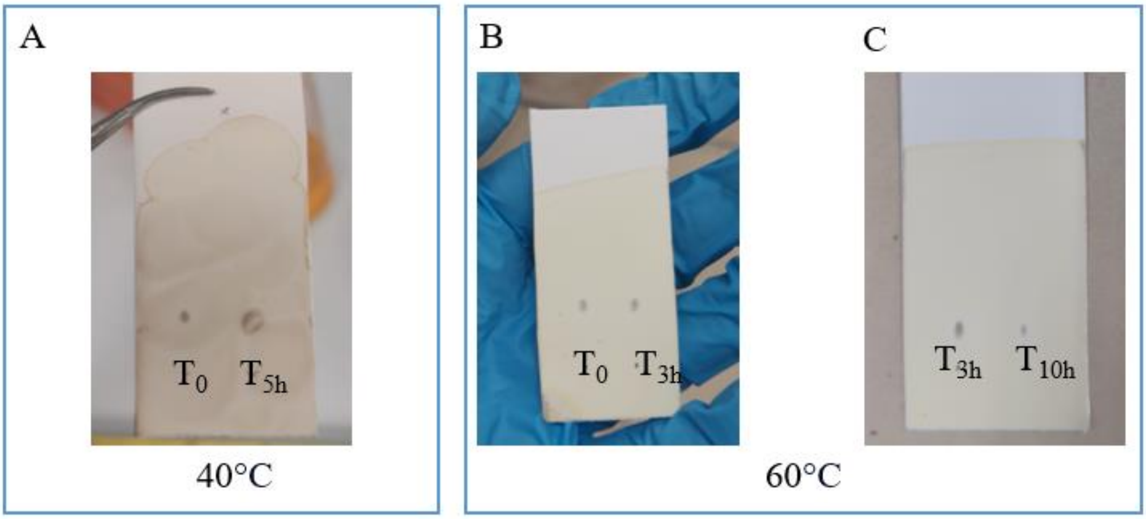

Fig. S2 TLC and cerium (IV) sulfate assay of ATAG samples: A) collected at 0 hours (T0, as control) and 5 hours (T_{5h}) at 40 °C, and B) collected at 0 hours (T0, as control), 3 hours (T_{3h}) and 10 hours (T_{10h}).

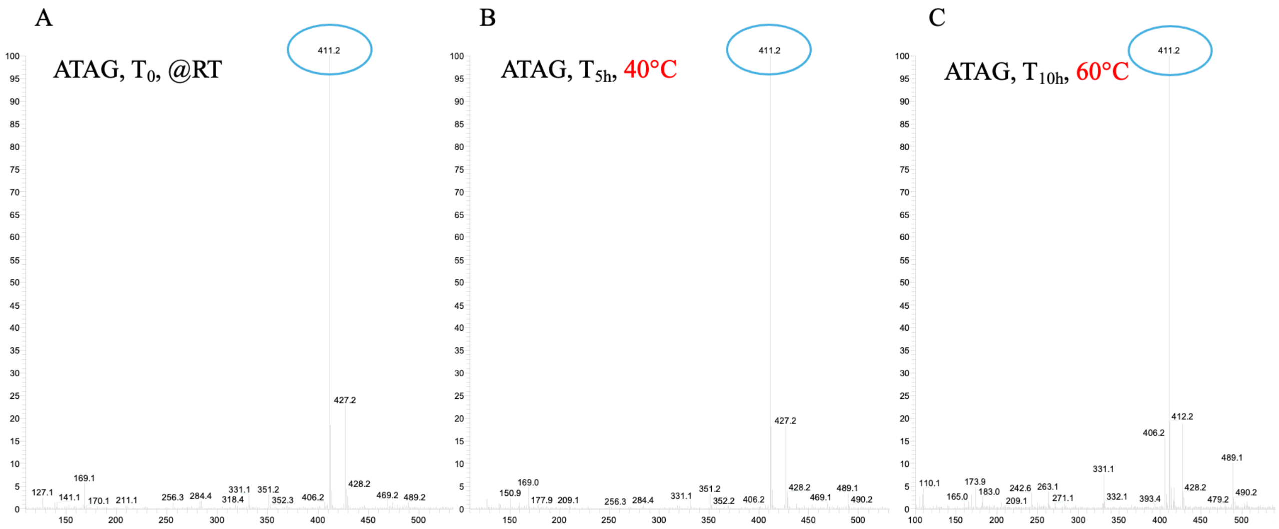

Fig. S3 Mass spectrometry spectra of ATAG samples collected at A) 0 hours (T0, as control) at RT, B) at 5 hours (T_{5h}) at 40 °C, and C) at 10 hours (T_{10h}) at 60 °C.

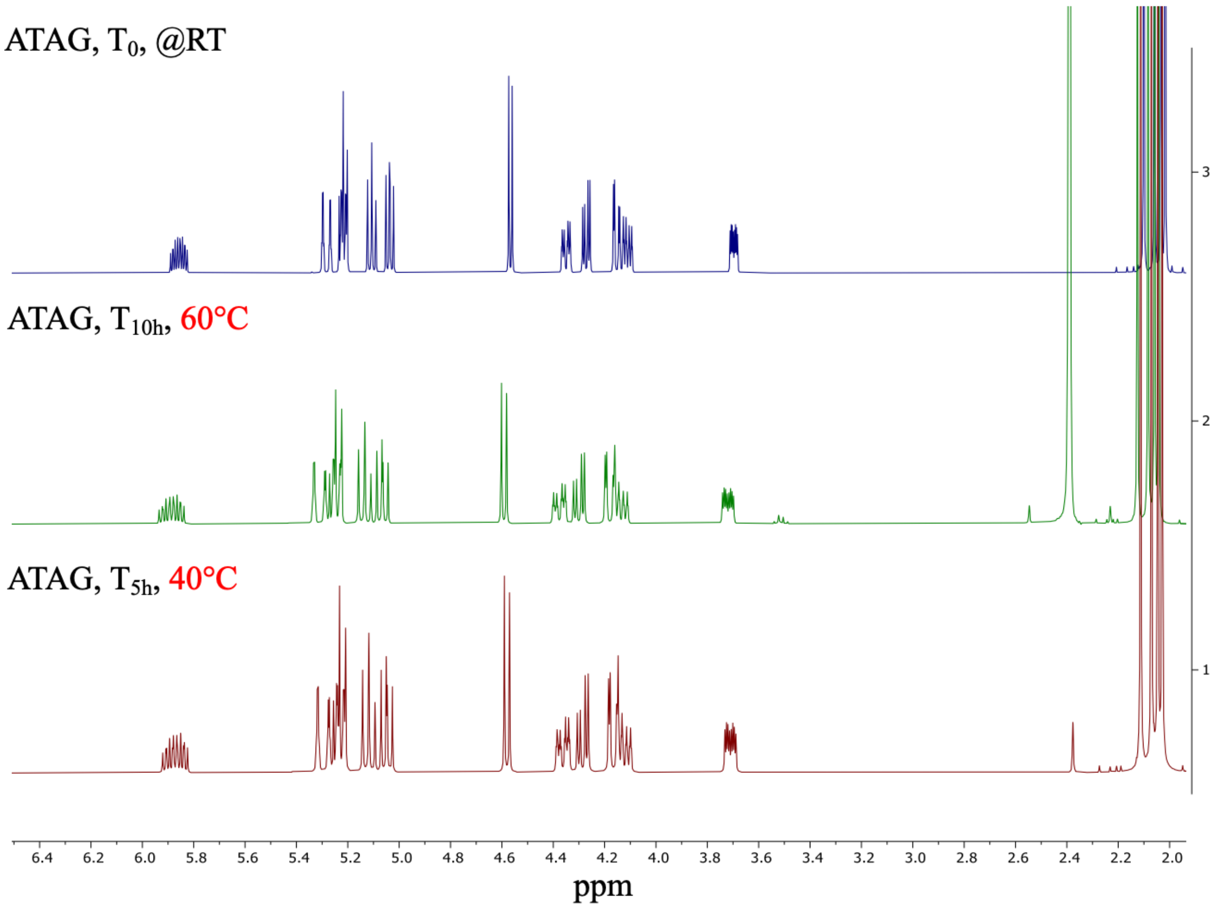

Fig. S4 Superimposition of spectra of ATAG samples collected at 0 hours (T0) at RT (in blue), at 10 hours (T_{10h}) at 60 °C (in green), and at 4 hours (T_{4h}) at 40 °C (in magenta).

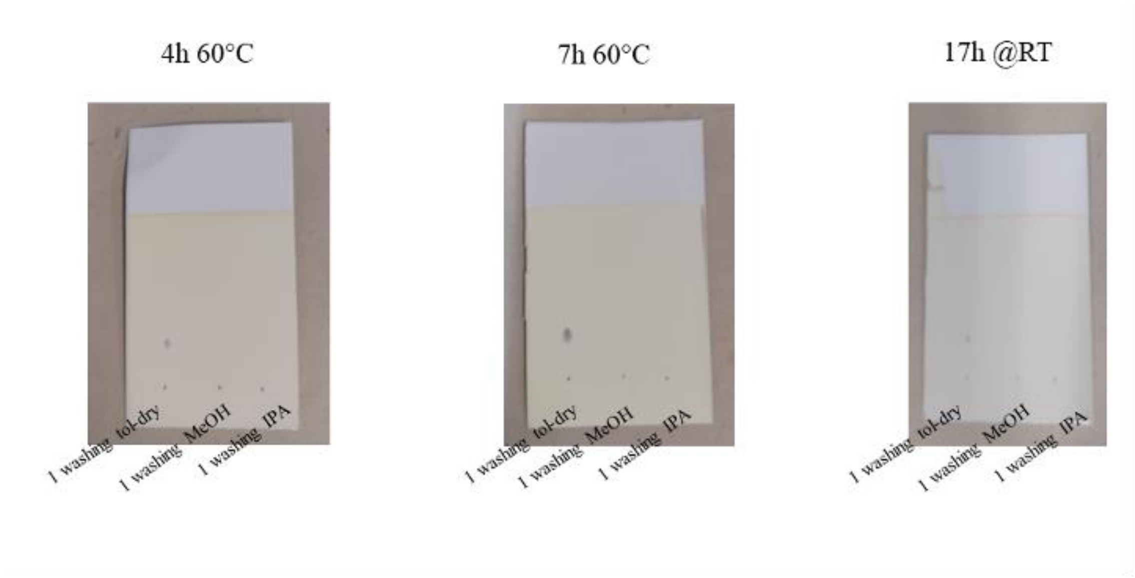

Fig. S5 TLC and cerium (IV) sulfate assay of washing supernatants collected at 4 hours (T_{4h}) and 7 hours (T_{7h}) at 60 °C, and at 17 hours (T_{17h}) at RT.

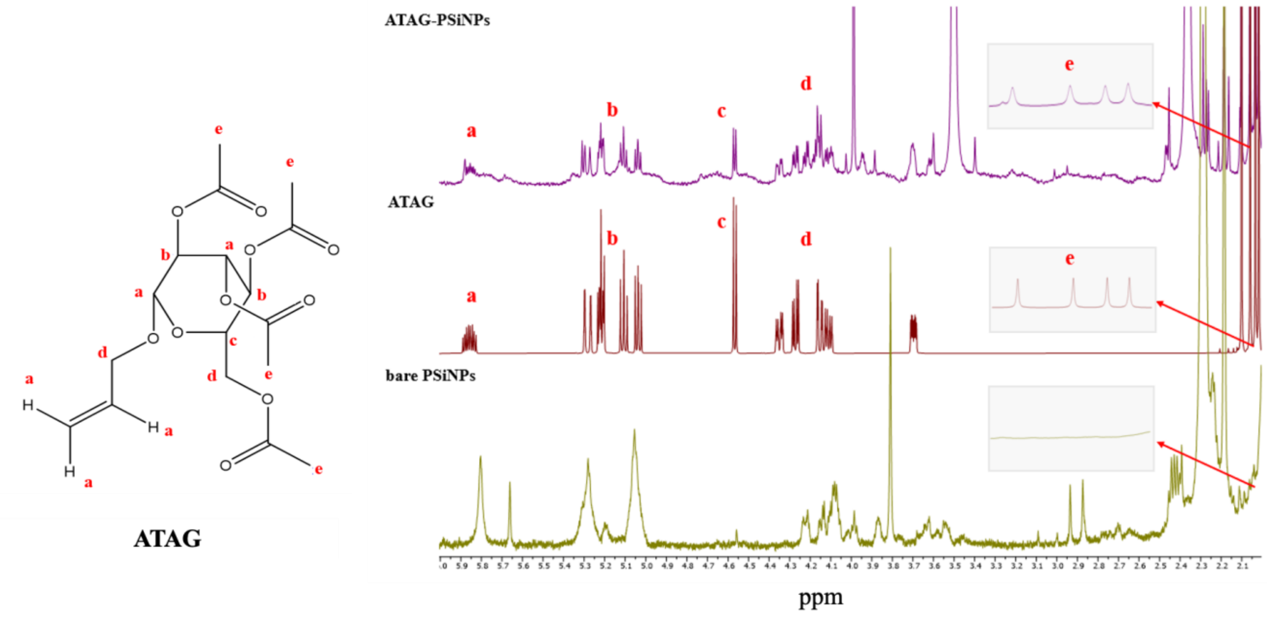

Fig. S6 Superimposition of NMR spectra of bare PSiNPs (in green), ATAG (in magenta), and ATAG-sPSiNPs (in violet). Spectra show the characteristic peaks of the Al-s moiety: Ha 5.31-6-07 ppm, Hb 5.26-5.56 ppm, Hc 4.55 ppm, Hd 4.0-4.38 ppm, He 2.02-2.04 ppm.

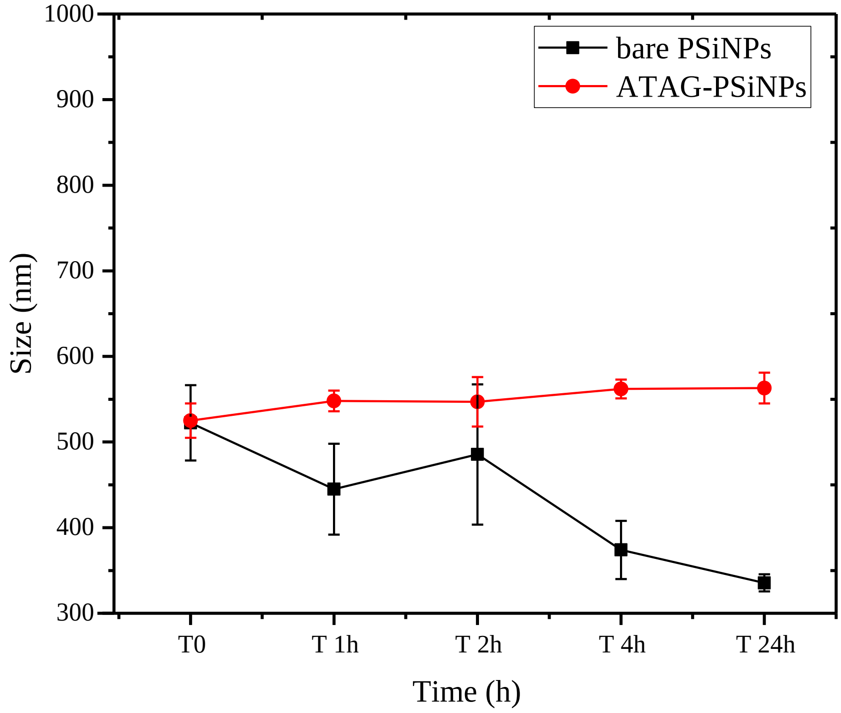

Fig. S7 Hydrodynamic diameter of bare PSiNPs (black) and ATAG-functionalized PSiNPs (red) measured after incubation in PBS (pH 7.2) for up to 24 hours at 37 °C. The error bars represent the standard deviation (SD) of three independent measurements (n=3).
